# Iron in Lung Pathology

**DOI:** 10.3390/ph12010030

**Published:** 2019-02-15

**Authors:** Vida Zhang, Elizabeta Nemeth, Airie Kim

**Affiliations:** 1 Department of Molecular and Medical Pharmacology, David Geffen School of Medicine at the University of California, Los Angeles, CA 90095, USA; vzhang@mednet.ucla.edu; 2 Department of Medicine, David Geffen School of Medicine at the University of California, Los Angeles, CA 90095, USA; enemeth@mednet.ucla.edu

**Keywords:** hepcidin, iron, lung, acute lung injury, COPD, lung infection, cystic fibrosis

## Abstract

The lung presents a unique challenge for iron homeostasis. The entire airway is in direct contact with the environment and its iron particulate matter and iron-utilizing microbes. However, the homeostatic and adaptive mechanisms of pulmonary iron regulation are poorly understood. This review provides an overview of systemic and local lung iron regulation, as well as the roles of iron in the development of lung infections, airway disease, and lung injury. These mechanisms provide an important foundation for the ongoing development of therapeutic applications.

## 1. Introduction

Iron is an essential trace mineral for normal biological function in almost all organisms. Most of the body’s iron is contained within the heme of hemoglobin, the vital oxygen carrier in blood. Iron is also required for cell viability and proliferation as a catalytic constituent of iron-containing proteins that are involved in DNA synthesis and repair, and cellular energy metabolism [1]. As expected, iron deficiency results in the impairment of both systemic oxygen delivery and cellular function. Conversely, an excess of iron also has deleterious effects for the host, leading to cellular toxicity via iron-generated oxyradicals and peroxidation of lipid membranes [2]. Systemically, increased iron availability is also associated with the increased virulence of multiple pathogens, including *Yersinia enterocolitica* [3], *Escherichia coli* [4], and *Klebsiella pneumoniae* [5]. In order to maintain an appropriate iron balance, organisms have evolved complex systemic homeostatic and cellular iron transport mechanisms [6].

Iron homeostasis in the lung faces unique challenges. The entire lung epithelium is exposed to inhaled air containing iron particulate matter and infectious pathogens, and is also part of a delicate air–blood interface whose gas exchange function is highly susceptible to impairment by cytotoxic injury. Thus, lung iron bioavailability must be highly regulated to prevent its use by microbes during infection and to ensure sequestration of catalytically active iron to prevent cytotoxicity. The terminal respiratory unit, the alveolus, is composed of three major cell types, all of which are active in the maintenance of lung iron homeostasis: types 1 and 2 alveolar epithelial cells, and alveolar macrophages. Alveolar macrophages are a specialized subset of macrophages that defend against pulmonary infections, and mediate damage and repair of the lung parenchyma [7]. However, the specific roles of these cell types in basal iron regulation or in response to injury or infection is still poorly understood. The purpose of this review is to explore recent scientific advances in understanding the role of iron regulation in lung pathologies.

## 2. Iron Regulation

### 2.1. Systemic Iron Homeostasis

A human adult requires ~25 mg/day of iron for baseline homeostasis and the replacement of minor unregulated iron losses. The majority of this iron comes from the recycling of senescent erythrocytes, while 1–2 mg is obtained from the absorption of dietary iron in the form of heme or non-heme iron [8]. During times of stress erythropoiesis, iron utilization by the bone marrow can increase 10-fold to accommodate the increased hemoglobin synthesis [9]. Thus, rapidly acting compensatory mechanisms have evolved to increase dietary iron absorption and to allow the rapid mobilization of iron from stores.

Hepcidin, a 25 amino acid peptide hormone produced primarily by hepatocytes [10], is the key regulator of systemic iron homeostasis. Hepcidin acts by binding to the transmembrane protein ferroportin (Fpn), the only known cellular iron exporter [11], causing its internalization and degradation within lysosomes [11,12]. As Fpn is highly expressed on duodenal enterocytes, macrophages, and hepatocytes, hepcidin controls the flow of iron from dietary gut absorption, recycling of erythrocytes, and tissue iron stores. Hepcidin production is stimulated by increases in plasma iron or iron stores, and during times of inflammation [13,14]. 

In addition to the mechanisms controlling systemic iron availability, each cell possesses regulatory mechanisms to coordinate its iron uptake, storage, and export. Most cells acquire iron by importing transferrin bound iron from blood via the membrane transferrin receptor (TfR1), after which iron is used for basal cellular requirements or stored in the form of ferritin. Splenic and hepatic macrophages also acquire iron through the phagocytosis of damaged or senescent erythrocytes, and this iron is similarly stored as ferritin or utilized for basic cellular functions [15]. Cellular iron export occurs through Fpn, which allows cells such as duodenal enterocytes and macrophages to release iron into circulation and maintain systemic iron homeostasis. In addition, Fpn expression is increased in iron-overloaded tissues and acts as a safety valve to export excess cellular iron to prevent oxidative damage. 

Coordination of cellular iron acquisition and distribution is regulated post-transcriptionally in response to changes in intracellular iron levels by the iron regulatory protein/iron responsive elements (IRP/IRE) system [16,17,18]. The iron regulatory proteins, IRP1 and IRP2, bind to IREs, which are untranslated regions of mRNA located at either the 5′ or 3′ end. IREs at the 5′ end are associated with genes involved in the storage or export of iron (ferritin, Fpn), while 3′ IREs are associated with genes involved in iron uptake (TfR1, DMT1). Under conditions of cellular iron depletion, IRP1/IRP2 bind to IREs, preventing translation of mRNA containing 5′ IREs and stabilizing mRNA containing 3′ IREs. This leads to the increased expression of iron uptake proteins and decreased expression of iron storage and export proteins. Conversely, in iron-loaded cells, IRP1 is converted to c-aconitase and IRP2 is degraded, resulting in decreased IRP binding to IREs. This leads to increased expression of iron storage and export proteins and decreased expression of iron uptake proteins.

### 2.2. Lung Iron Regulation

**Figure 1:**
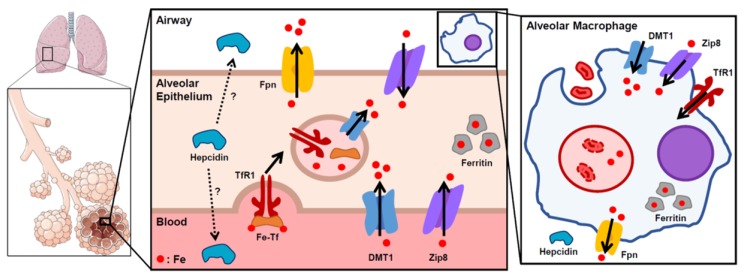
Proposed scheme of lung iron homeostasis. Iron is taken up into the alveolar epithelial cells through transferrin receptor (TfR1) and DMT1, and exported through ferroportin (Fpn), which was reported to be localized on the apical/airway facing layer of the epithelium. Within the cells, iron is stored in a non-reactive state in ferritin. Though hepcidin is mostly produced in the liver for systemic circulation, local production of hepcidin has also been suggested to play a role in lung iron homeostasis. Zip8 is highly expressed in the lung and facilitates iron intake, though its specific distribution remains unknown. Inset depicts an alveolar macrophage. Macrophages phagocytose airway red blood cells to recycle iron from heme. Solid arrows indicate direction of iron transport.

Iron regulation in the lung has not been well characterized, with only a few in vitro and in vivo studies attempting to identify the iron transporters in pulmonary cells (Figure 1). TfR1 has been identified as an importer of transferrin-bound iron in the alveolar epithelium and alveolar macrophages. In response to systemic iron deficiency, TfR1 protein levels increased in whole rat lung. Conversely, TfR1 protein expression in whole lung did not increase with intratracheally instilled iron oxide [19]. The DMT1 transporter, a principal importer of dietary non-heme iron and an importer of endosomal iron from the Tf-TfR1 complex into cytoplasm, has also been shown to be expressed in both alveolar epithelial cells and alveolar macrophages [20]. While respiratory DMT1 appears to be regulated by the IRP/IRE system, there is inconclusive evidence on the effects of iron deficiency and overload on the production of DMT1 mRNA and protein in the lung [19,21,22]. Multiple studies utilizing DMT1 mutated murine models have implicated this iron transporter in the pathogenesis of lung injury. The Belgrade rat, an animal model of functional DMT1 deficiency, develops more severe lung injury in response to lipopolysaccharide (LPS) and oil fly ash [23,24]. Similarly, *mk*/*mk* mice, also defective in DMT1, have increased bleomycin-induced lung injury compared to wild-type controls [25]. The mechanism of DMT1 attenuating the lung’s response to inflammatory stimuli is unclear, but these descriptive studies demonstrate a link between DMT1 and the lung’s inflammatory response. Thus, although iron importers are present in the lung, little is understood about their regulation and role in specific cell types and under different pathophysiological conditions.

Fpn was reported to be localized on the apical layer of the airway epithelia of human lung [26] and also expressed in alveolar macrophages [27]. While the liver is the predominant source of circulating hepcidin, the hormone is also expressed at lower levels in human airway epithelial cells and alveolar macrophages [28], raising the possibility that hepcidin has a paracrine role in the lung. One ex vivo study showed that LPS-stimulated mouse alveolar macrophages increased the expression of hepcidin mRNA and decreased *Fpn* mRNA. Iron treatment had no effect on hepcidin mRNA expression, but did upregulate both *Dmt1* and *Fpn* [29]. Interestingly, another in vitro study showed that Fpn in airway epithelial cells did not internalize in response to hepcidin and that hepcidin itself had no significant effect on iron transport in either airway epithelial cells or alveolar macrophages [30]. However, Fpn levels do correlate with changes in iron status [27,31], suggesting that lung Fpn expression may be controlled preferentially by the IRP/IRE regulatory system, rather than a purely hepcidin-dependent mechanism. Additionally, the reported distribution pattern of Fpn on the apical layer of airway epithelial cells suggests that Fpn may play a tissue-specific role of iron detoxification in lungs [26].

Recent studies in mouse models with genetic iron overload have begun characterizing the roles of hepcidin and Fpn in lung iron homeostasis. Neves et al. investigated a murine disease model of hereditary hemochromatosis type 4, created by a global C326S amino acid substitution in Fpn that confers resistance to hepcidin binding and leads to systemic iron overload [32]. They reported that the Fpn mutant mice develop increased iron levels in airway epithelial cells and bronchoalveolar lavage (BAL) fluid [27]. Interestingly, while splenic and hepatic macrophages are predictably iron-depleted from the increased Fpn protein exporting iron, a subset of alveolar macrophages showed iron overload. The authors suggest that these differences could be partially due to the varying levels of Fpn expression in alveolar macrophages. 

Deschemin et al. characterized the lungs in hepcidin knockout (HKO) mice, a mouse model of severe genetic iron overload [31]. The lack of hepcidin results in increased gut iron absorption with systemic iron overload, iron deposition in parenchymal tissues, and iron depletion of macrophages in the liver and spleen. Iron content in the lung increased, which resulted in compensatory decreases in *Tfrc* and *Dmt1* mRNA expression, and increased levels of Fpn and ferritin mRNA and protein levels. Specific examination of the alveolar macrophages also demonstrated increased levels of Fpn and ferritin. However, similar to the paper by Neves et al., these macrophages were iron-loaded, which is in stark contrast to the iron depletion of splenic macrophages. This is likely related to the much lower expression of Fpn in the alveolar macrophages of HKO mice compared to the splenic macrophages. While these genetic mouse models of iron overload clearly demonstrate some unique characteristics of lung iron homeostasis, further studies are necessary to clarify the role of the hepcidin–Fpn axis in the lung.

ZIP8, encoded by the *SLC39A8* gene, is a member of the SLC39A transmembrane metal importer family and is expressed many-fold higher in the lung than in any other organ system [33]. Initially identified as a zinc transporter, recent studies have shown that Zip8 also imports iron into the cytosolic space [33]. Early in vitro and animal studies consistently show that inflammatory stimulation with LPS greatly increases Zip8 expression, suggesting that this iron importer has a function in host defense [34,35]. As of yet, there have been no studies investigating the role of Zip8 in human lung infections or lung injury. 

## 3. Iron in Lung Pathology

### 3.1. Acute Lung Injury

Acute lung injury (ALI), clinically known as acute respiratory distress syndrome (ARDS), is an acute inflammatory process with neutrophil infiltration, increased vascular permeability, and diffuse alveolar damage [36]. ARDS can occur as a result of a direct insult to the lung, including pneumonia, aspiration, and smoke inhalation, or a systemic inflammatory response, including sepsis, trauma, burns, and transfusion-related ALI. The pathophysiology of ARDS is largely mediated by the release of free radicals and reactive oxygen species and their injurious effects on endothelial integrity [37]. In the presence of iron, peroxides are converted to damaging radicals and enhance cytotoxicity by the Fenton reaction [38]. The relevance of iron in the development of ARDS has been evaluated with numerous clinical studies, showing strong correlations with iron and iron-related proteins, as well as the presence or severity of ARDS. One study found increased concentrations of BAL total iron and non-heme iron in ARDS patients, as compared to healthy controls. Iron-related proteins, including hemoglobin, transferrin, TfR1, lactoferrin, and ferritin, were also all elevated in BAL [39]. Future studies are needed to examine whether these changes in BAL iron and iron-related proteins have a causal effect on lung injury or are simply a byproduct of lung injury and increased vascular permeability. Serum ferritin has also been investigated as a predictor of ARDS, and was found to predict the development of ARDS with high sensitivity and specificity in one clinical study [40]. As ferritin is a known acute phase reactant and could simply be rising as part of the inflammatory response, a second clinical study confirmed the predictive value of serum ferritin for the progression to ARDS while also demonstrating no correlation of ferritin with the degree of hypoxia, time of invasive ventilation, or mortality [41]. These studies consistently illustrate a strong association between iron-related proteins and the development of lung injury, but further clinical and basic mechanistic studies are necessary to delineate the causative effects of iron on ARDS.

### 3.2. Lung Infections

Host defense of the lung organ system is particularly challenging as the entire epithelium is in constant and direct contact with environmental air containing numerous potential infectious pathogens. As the delicate single-cell-layer alveolar epithelium is responsible for vital air–blood gas exchange, non-cytotoxic nutrient deprivation mechanisms of host defense play an important role against lung microbial pathogens. The biological relevance of iron in the pathology of infections has been established through numerous clinical and animal studies. Iron overload has been associated with increased incidence of bacteremia with hemodialysis [42], and increased virulence of multiple microbes, including *Yersinia enterocolitica* [3], *Escherichia coli* [4], and *Klebsiella pneumoniae* [5]. One clinical study found a significant correlation between high dietary iron and the development of active tuberculosis in a high-risk population [43]. Another study of 137 iron-deficient Somali patients found that iron repletion resulted in a significant increase in infection incidence, with the activation of pre-existing malaria, brucellosis, and tuberculosis [44]. 

**Figure 2:**
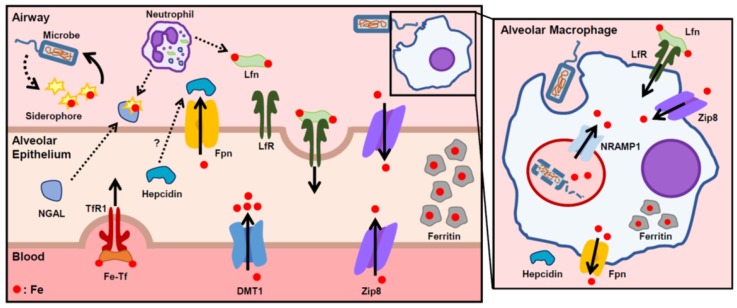
Proposed scheme of lung iron regulation during infection and inflammation. Bacteria secrete siderophores to capture host iron. The host combats this by increasing import of iron (lactoferrin (Lfn), DMT1), decreasing export of iron through ferroportin (Fpn), and increasing iron stores through ferritin. Secreted Lfn and NGAL bind free iron and siderophore-bound iron in the airway to prevent bacterial iron uptake. Inset shows an alveolar macrophage during infection. Alveolar macrophages phagocytose bacteria as a host defense response. NRAMP1 is expressed in macrophage phagosomes and transports iron out of the phagosome to limit iron availability to pathogens. Solid arrows indicate direction of iron transport.

Both invading pathogens and their hosts have developed multiple mechanisms to control the supply of iron necessary for microbial survival (Figure 2). One particularly effective and well-described method of iron scavenging by microbes is through the siderophore-dependent pathways, in which microbes secrete small compounds called siderophores that complex with iron for active uptake by the microbe [45]. In response, the host circumvents this pathway with neutrophil gelatinase-associated lipocalin (NGAL), otherwise known as siderocalin or lipocalin-2. Produced by neutrophil granules and epithelial cells in response to inflammation, this innate immune protein acts by binding to, and sequestering, iron-loaded bacterial siderophores [46]. One murine study showed that intratracheal *Escherichia coli* instillation resulted in a strong induction of NGAL expression in bronchial epithelium and type 2 pneumocytes [47]. An in vitro study of *Mycobacterium tuberculosis* found that recombinant NGAL restricted the growth of the organism in broth media in an iron-dependent manner [48]. Another major iron-binding protein in the airways is lactoferrin, a member of the transferrin gene family that is found in nasobronchial epithelial secretions and neutrophil granules. Lactoferrin sequesters airway iron away from microbes and is taken up by the lactoferrin receptor on lung epithelial cells and macrophages for iron reabsorption. Levels of lactoferrin correlate with the severity of infectious pneumonia, pulmonary tuberculosis, and sepsis [49]. Another iron transporter that is vital in host defense is natural resistance-associated macrophage protein 1 (NRAMP1), a divalent metal transporter expressed specifically in phagosomes. NRAMP1 reduces phagosomal iron availability and confers resistance to several intraphagosomal microbes [50,51].

The hepcidin–Fpn axis also has a principal role in innate immunity. Hepcidin plays a role in host defense by sequestering iron to hinder the growth and proliferation of invading pathogens [52]. During times of infection and inflammation, there appears to be multiple mechanisms of hepcidin expression regulation. The primary regulator of inflammation-induced hepcidin production is the inflammatory cytokine IL-6. A single infusion of IL-6 into healthy human subjects increased hepcidin production and decreased serum iron [13]. In an inflammatory state, increased IL-6 causes the activation of STAT3 (signal transducer and activator of transcription 3) and its binding to the hepcidin promoter [53]. In response to bacterial stimulation, myeloid cells have also been shown to upregulate hepcidin and decrease Fpn expression in a Toll-like receptor 4 (TLR4)-dependent manner [54].

Recently, several transgenic murine studies have established the roles of hepcidin and iron status in the morbidity and mortality of various pathogenic infections. Hepcidin knockout mice developed hyperferremia with a profound susceptibility to bacteremia from *Klebsiella pneumoniae*, *Yersinia enterocolitica*, and *E. coli*, and treatment with a hepcidin analogue restored hypoferremia, decreased bacterial burden, and improved survival in each model of infection [5,55,56]. These studies indicate that hepcidin has a protective effect against siderophilic pathogens by limiting the availability of non-transferrin bound iron, a form of iron that is readily accessed by microbes. Another study showed that cell-specific knockdown of hepcidin in airway epithelium increased lung bacterial burden and injury in mice after cecal ligation and puncture, a model of polymicrobial sepsis [57]. By contrast, hepcidin–Fpn regulation may have a deleterious effect on the host during infections with intracellular pathogens. For example, one in vitro study demonstrated that the intracellular growth of *Chlamydia psittaci*, *C. trachomatis*, and *Legionella pneumophila* in macrophages is enhanced by the addition of hepcidin. Accordingly, macrophages from flatiron mice, a mouse strain heterozygous with a loss-of-function Fpn mutation (H32R), had increased bacterial proliferation that was unchanged by the addition of exogenous hepcidin [58]. Another study used murine macrophages to show decreased virulence of intracellular *Salmonella enterica* when Fpn expression was increased, and increased virulence with hepcidin-induced Fpn downregulation [59]. 

### 3.3. Cystic Fibrosis

Cystic fibrosis (CF) is a genetic disorder caused by mutations in the cystic fibrosis transmembrane conductance regulator (CFTR) gene, and is characterized by the retention of thick airway secretions and chronic pulmonary infections. Patients with CF also have increased levels of iron and iron-related proteins in their lower respiratory tract [60], and these iron levels are strongly correlated with increases in inflammatory cytokines [61]. A clinically important area of study in CF is the microbial pathogen *Pseudomonas aeruginosa*, which is responsible for the majority of CF infectious exacerbations. This pathogen forms biofilms that are more resistant to antibiotics than the free-floating (planktonic) state and hinder the eradication of these bacteria. Construction of this biofilm is highly dependent on iron availability, and *Pseudomonas aeruginosa* has developed sophisticated mechanisms to acquire iron from the environment, including both siderophore-dependent and independent systems [62]. 

### 3.4. Chronic Obstructive Pulmonary Disease

Both cigarette smoking and chronic obstructive pulmonary disease (COPD) are associated with a disruption of iron homeostasis in the lung. Regular cigarette smoking results in a great increase in lung exposure to iron, estimated at 5.2–13.8 µg of iron daily in a subject smoking 20 cigarettes per day [63]. Compared to nonsmokers, smokers have increased total non-heme iron and ferritin levels in BAL fluid and in alveolar macrophages [64], as well as increased serum ferritin levels [65]. Smokers also have increased redox-active iron levels in exhaled breath condensate [66]. 

Although cigarette smoking is the largest risk factor for the development of COPD, genetic association studies have uncovered differentially expressed iron-related genes that indicate a role for iron in the susceptibility to developing COPD. The most relevant is the iron-regulatory protein IRP2 that was discovered to be a major COPD susceptibility gene in a case–control study [67]. Notably, IRP2 is located within a cluster of genes on a chromosome that also includes several components of the nicotinic acetylcholine receptor [68]. As in smokers, levels of iron and iron-binding proteins are increased in the lung tissue, BAL fluid, and alveolar macrophages of COPD patients, and iron levels are correlated with disease severity and with worsening lung function [64]. A recent study used mouse models of COPD to investigate the mechanism of IRP2’s role in the development of COPD [69]. The group showed that IRP2 increases mitochondrial iron loading and levels of cytochrome c oxidase (COX), leading to mitochondrial dysfunction and COPD development. Frataxin-deficient mice, which develop higher mitochondrial iron loading due to lack of the mitochondrial iron-sulfur regulator frataxin, were shown to develop worse airway mucociliary clearance and higher pulmonary inflammation at baseline. Conversely, mice with decreased COX synthesis were protected from cigarette smoke-induced lung inflammation and impairment in mucociliary clearance. Mice treated with the mitochondrial iron chelator deferiprone were protected from impaired mucociliary clearance, pulmonary inflammation, and the development of COPD. Together, these data indicate that IRP2 functions as a COPD susceptibility gene by increasing mitochondrial iron overload and levels of COX, leading to mitochondrial dysfunction and the development of COPD.

## 4. Potential Therapeutics

Due to the apparent importance of iron homeostasis in the development of many lung pathologies and infections, there has been a lot of interest in manipulating iron availability for potential therapeutic applications. Conventional means of reducing iron load in the body include dietary restriction [70], phlebotomy, and chelators, including deferoxamine, deferiprone, and deferasirox [71]. However, such systemic iron depletion can predispose patients to multiple adverse effects, including nutritional deficiency, anemia, and infection. For example, systemic iron chelators such as deferoxamine have been shown to eliminate *Pseudomonas aeruginosa* biofilms on a CF line in vitro [72], but deferoxamine also functions as a bacterial siderophore and can paradoxically act as an iron supply to specific microbes such as *Rhizopus* [73]. Newer synthetic iron chelators with fewer adverse effects are also being considered for a broad range of oxidative stress-related conditions, ranging from cardiovascular to inflammatory and malignant pathologies [74]. In addition, there has been early work examining the therapeutic potential of modulating specific iron transporters, including DMT1 and Fpn [12,75]. Studies in mice have shown that hepcidin mimetics are effective in lowering systemic iron and modulating the virulence and mortality of gram-negative pneumonia [5,76]. Another approach in the field of CF research is the use of an iron competitor. Interfering with iron uptake by *Pseudomonas aeruginosa*, with the cationic metal gallium, has been shown to have antimicrobial and antibiofilm activity [77]. Another proposed, but undeveloped, therapeutic approach would be the local or regional delivery of iron modulators, such as chelators, iron competitors, or hepcidin analogues. 

While there have been substantial scientific advances in our understanding of systemic iron regulation and the pathogenesis of different iron disorders in recent years, lung iron regulation and its role in pulmonary pathology has been an understudied area. Future basic and translational studies are clearly necessary to advance this field and to enable the development of clinical therapeutic applications. On a mechanistic level, the field requires a systematic characterization of the roles and regulation of the principal iron transporters and iron-related proteins in each of the major alveolar cell types as well as the bronchial airway epithelium. Transgenic mice with lung-specific deletions of iron transporters, and further mouse models of key pulmonary pathologies, could be utilized to study the roles of specific iron-related proteins in the development of lung disease.
